# Flecainide acetate inhalation solution for cardioversion of recent-onset, symptomatic atrial fibrillation: results of the phase 3 RESTORE-1 trial

**DOI:** 10.1093/europace/euaf064

**Published:** 2025-03-25

**Authors:** Michiel Rienstra, Anderson C Woite-Silva, Aaf Kuijper, Sabine Eijsbouts, Karin Kraaier, Tomas Janota, Clara Van Ofwegen, Ype Tuininga, Erik Badings, Jose Luis Merino, Jeremy N Ruskin, A John Camm, Peter R Kowey, Christopher Dufton, Jean Maupas, Dawn Parsell, Luiz Belardinelli

**Affiliations:** Department of Cardiology, University of Groningen, University Medical Center Groningen, Hanzeplein 1, 9713 GZ Groningen, The Netherlands; InCarda Therapeutics, Newark, CA, USA; Department of Cardiology, Spaarne Gasthuis, Haarlem, The Netherlands; Department of Cardiology, Maxima Medical Center, Veldhoven, The Netherlands; Department of Cardiology, Medical Center Leeuwarden, Leeuwarden, The Netherlands; Department of Cardiology, General University Hospital, Prague, Czechia; Department of Cardiology, Diakonessenhuis, Utrecht, The Netherlands; Department of Cardiology, Deventer Hospital, Deventer, The Netherlands; Department of Cardiology, Deventer Hospital, Deventer, The Netherlands; Department of Cardiology, La Paz University Hospital, IdiPaz, Universidad Autonoma, Madrid, Spain; Cardiac Arrhythmia Service, Massachusetts General Hospital, Boston, MA, USA; Cardiovascular and Genetics Research Institute, St. George’s University of London, London, UK; Division of Cardiovascular Diseases, Lankenau Heart Institute, Wynnewood, PA, USA; InCarda Therapeutics, Newark, CA, USA; InCarda Therapeutics, Newark, CA, USA; InCarda Therapeutics, Newark, CA, USA; Parsell Consulting, Cedar Park, TX, USA; InCarda Therapeutics, Newark, CA, USA

**Keywords:** Atrial fibrillation, Cardioversion, Flecainide, Oral inhalation

## Abstract

**Aims:**

Atrial fibrillation (AF) is the most prevalent cardiac arrhythmia. New treatments are needed to cardiovert recent-onset paroxysmal AF quickly and safely. RESTORE-1 was a multicentre, randomized, double-blind, placebo-controlled trial of a 120 mg orally inhaled solution of flecainide acetate (FlecIH-103) for cardioversion of symptomatic, recent-onset (≤48 h) paroxysmal AF. The study aim was to evaluate the efficacy and safety of FlecIH-103 administered via oral inhalation.

**Methods and results:**

Patients experiencing a recent-onset paroxysmal AF episode were randomized to receive a single dose of FlecIH-103 or placebo delivered over two 3.5 min inhalation periods, while patients were monitored using 12-lead electrocardiograms and Holter. The trial was stopped prematurely after treating 55 patients, due to lower-than-expected conversion rates and plasma levels. Mean age was 59.6 years, 31.5% of patients were female, and 59.2% were having their first AF episode. Conversion rate was 30.8% (95% confidence interval: 14.7–43.8) for the active group (*n* = 39) and 0.0% for the placebo group (*n* = 12) (*P* = 0.04). Median time to conversion was 12.8 min (IQR: 17.2). In the active group, the mean flecainide plasma level was 198 ng/mL (SD: 156), which is ∼50% lower than in the previous studies. The most common adverse events (AEs) were dysgeusia, dyspnoea, and cough. All AEs were short-lasting and of mild or moderate intensity.

**Conclusion:**

Despite early termination of the trial, FlecIH-103 was significantly more effective than placebo in cardioverting AF. Safety data did not show any serious AEs. Further studies of FlecIH-103 are needed to optimize the combination of drug formulation and inhalation delivery platform.

**Clinical trial registration:**

URL: https://clinicaltrials.gov, unique identifier: NCT05039359

## Graphical Abstract

**Graphical Abstract:**
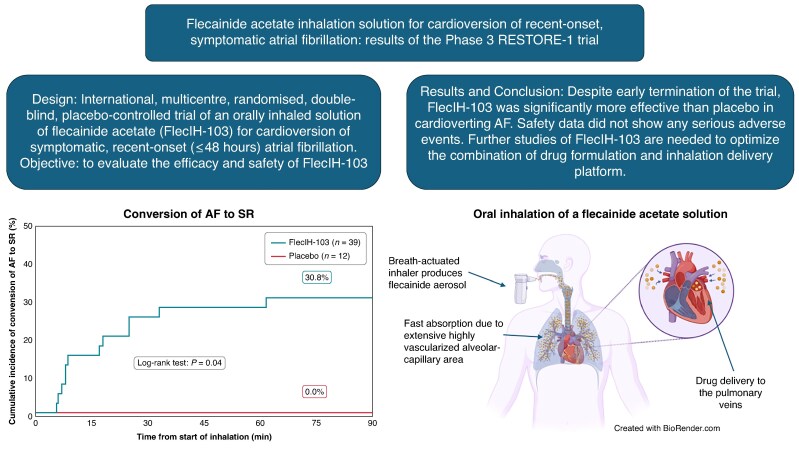

**What’s new?**

- The randomized, double-blind, placebo-controlled RESTORE-1 clinical trial assessed the efficacy and safety of an orally inhaled solution of flecainide acetate (FlecIH-103).
- FlecIH-103 cardioverted significantly more patients with recent-onset (≤48 h) paroxysmal atrial fibrillation (AF) from AF to sinus rhythm (SR) than placebo, with no serious adverse event reported.
- Implementation of subsequent therapies in patients whose AF did not convert to SR was not delayed by receiving FlecIH-103. Likewise, time to discharge from the Emergency Department was not impacted by receiving FlecIH-103.

## Introduction

Atrial fibrillation (AF) is a worldwide health burden that is associated with significantly disabling complications, such as stroke and heart failure (HF).^1^ The pathophysiology of incipient AF, often presenting in paroxysmal episodes, is thought to be related to arrhythmogenic activity arising from the pulmonary vein sleeves.^2^ There has been renewed interest in rhythm control as first-line treatment for AF because early restoration of sinus rhythm (SR) upon detection of AF has been associated with a reduction in adverse cardiovascular events.^3,4^

Orally inhaled flecainide (FlecIH) is being developed for cardioversion of recent-onset (≤48 h) paroxysmal AF. The advantages of delivery of flecainide to the left atrium via the pulmonary veins through oral inhalation are more rapid onset of action and shorter time to conversion compared to oral administration (i.e. the pill-in-the-pocket approach) and, potentially, lower incidence of adverse events (AEs) compared to intravenous administration.^5–10^ We have previously reported the positive results of a Phase 2 study (INSTANT-1), wherein the efficacy and safety of FlecIH were described.^11–14^

For the current Phase 3 study (RESTORE-1), we sought to compare the efficacy and safety of FlecIH to a placebo inhalation solution in a randomized, double-blind trial. Upon blinded review of the data accumulated from the first 55 patients enrolled, the conversion rate from AF to SR was found to be lower (30.8%) than that observed in the INSTANT-1 trial (42.6%).^14^ Similarly, the mean peak plasma concentrations of flecainide were nearly two-fold lower than expected. These observations led to early termination of the trial for futility reasons, i.e. limited clinical relevance, and not due to safety.

## Methods

### Design

This was a Phase 3, multicentre, randomized, double-blind, placebo-controlled study designed to evaluate the efficacy and safety of flecainide acetate inhalation solution (FlecIH-103), compared with placebo in patients with recent-onset, symptomatic, newly diagnosed or paroxysmal AF (ClinicalTrials.gov: NCT05039359).

This study was conducted at 24 sites in Europe, Canada, and the USA. The study protocol was approved by independent ethics committees at each participating site. Patient safety was monitored by an independent data monitoring committee. Informed consent was obtained in writing from all participants prior to any study-related procedure or administration of FlecIH-103.

### Participants

The study population included adults between the ages of 18 and 85 years with a recent-onset, symptomatic, newly electrocardiogram (ECG)-diagnosed or recurrent episode of paroxysmal AF; recent-onset was defined as having a symptom duration of at least 1 h and less than or equal to 48 h at the time of dosing. The AF-related symptoms were defined as any of the following: heart palpitations, chest pain/pressure, dizziness/lightheadedness, or shortness of breath/dyspnoea.

Key exclusion criteria from the study included a history of persistent AF, haemodynamic instability of any sort, relevant structural heart disease [e.g. HF with reduced ejection fraction, with New York Heart Association (NYHA) Class III/IV symptoms, recent HF-related hospitalization, previous or current evidence of significant left ventricular dysfunction/hypertrophy], ongoing myocardial ischaemia, recent use (≤7 days) of Class I/III antiarrhythmic drugs (AADs), recent (≤12 weeks) use of amiodarone, or hypersensitivity to flecainide.

### Procedures

#### Screening period

After informed consent was obtained, patients were monitored using cardiac telemetry and a continuous ECG patch for at least 45 min prior to randomization. Further data obtained included a medical history, standard 12-lead ECGs, vital signs, targeted physical examination, and laboratory blood samples.

Eligible patients were then randomized in a proportion of 3:1 to receive FlecIH-103 at a total estimated total lung dose (eTLD) of up to 120 mg or a placebo inhalation solution. Randomization was stratified by geographic region (US and non-US) and duration of symptoms of the current AF episode (≥1 to ≤24 h and >24 to ≤48 h).

#### Observation period

Patients were continuously monitored via telemetric ECG and had their vital signs measured while inhaling FlecIH-103 or placebo and for 90 min thereafter. Dosing occurred over an 8 min inhalation period, comprising two 3.5 min inhalations periods separated by a 1 min break. Blood samples for measurement of plasma flecainide concentrations were obtained before and at 2 min and 90 min after completion of inhalation. Conversion of AF to SR was established during the observation period if SR was observed for at least 1 min.

Patients whose AF did not convert to SR during the observation period could be offered standard-of-care treatment, at the discretion of the investigator [e.g. electrical cardioversion (ECV) or pharmacological cardioversion (PCV)], except with QT-prolonging drugs, such as ibutilide, procainamide, and/or sotalol.

#### Follow-up period

After completion of the 90 min observation period, patients were discharged, and their post-discharge medical management was at the discretion of the investigator. Patients were considered ‘discharge-eligible’ when all of the following criteria were met: patient had completed the observation period (90 min time point); any AE had resolved or was considered stable; any AF-related symptoms had resolved or were indicated acceptable by the patient; patient’s heart rate/rhythm and vital signs had been stable for at least 30 min; and if the patient’s heart rhythm was not in SR, the patient had received or declined standard of care treatment(s). Patients were contacted at 24 h and 96 h after initiation of dosing for evaluation of AF recurrence, which was determined based on AF-related symptoms and clinical assessment (ECG was not obligatory).

### Endpoints

The primary efficacy endpoint was the proportion of patients whose AF converted to SR during the 90 min observation period. Secondary efficacy endpoints were the following: the time to conversion of AF to SR within the 90 min observation period; the proportion of patients with AF-related symptoms at the 90 min time point; the proportion of patients requiring hospitalization prior to discharge; the prevalence (i.e. events per patient) of additional AF-related interventions required prior to discharge; and the time to discharge-eligible status. The AF-related interventions were defined as ECV, non-study drug PCV, or new/increased rate control medication prior to discharge.

Safety endpoints included the proportion of patients experiencing treatment-emergent AEs (TEAEs); treatment-emergent serious AEs (SAEs); or treatment-emergent cardiovascular events of special interest (CV-AESIs). The CV-AESIs were defined as cardiovascular AEs related to flecainide, that is, hypotension, ventricular tachycardia, sinus pause/bradycardia, atrial flutter (AFL) with 1:1 conduction, and other arrhythmias. Safety endpoints also included the following: the change from baseline in QRS interval duration after completion of dosing; the proportion of patients with a markedly prolonged QRS interval (i.e. ≥ 130 ms) after completion of dosing; and the change in vital signs (HR, SBP, DBP, respiratory rate, and SpO_2_) from baseline to the 90 min time point.

### Statistical analysis

A sample size of approximately 400 patients was estimated to detect a difference between groups, with an estimated conversion rate ≥ 35% for the active group and ≤17% for the control group, at a 0.05 significance level, with ≥90% power. The same sample size was estimated to be sufficient to detect low-frequency safety events (e.g. 1% event rate).

The intention-to-treat population (ITT population) was composed of all patients who were randomized. The safety population included all patients who were exposed to the study drug. The efficacy population was defined post hoc as the patients who were in AF at the start of inhalation and who, after inhalation, had plasma levels of study drug consistent with their assigned treatment group. The pharmacokinetics (PK) population was defined as all patients randomized to receive FlecIH-103 who received the full dose of study drug and had detectable plasma flecainide concentrations after dosing. The efficacy population was used to assess efficacy endpoints, while the safety population was used to assess safety endpoints. The PK population was used for summarizing the estimated plasma concentration levels.

Quantitative data are reported as mean with standard deviation (SD) or with a 95% confidence interval (95% CI), with geometric mean and geometric coefficient of variation also reported for plasma PK concentrations. Categorical data are expressed in frequencies and percentages with a 95% CI using the Wilson method. The Student’s *t*-test was used to compare continuous variables, the Fisher’s exact test for categorical variables, and the log-rank test for time-to-event data. All comparisons and 95% CI were performed using a two-sided approach, and statistical significance was defined as *P* < 0.05. Adverse events are reported using the Medical Dictionary for Regulatory Activities (MedDRA; version 25.01). Statistical analyses were performed using SAS® software, version 9.4, and R, version 4.3.

## Results

The trial was prematurely stopped after treatment of 55 of the planned 400 patients, due to lower-than-expected conversion rates and flecainide plasma levels.

### Disposition (flowchart)

**Figure 1:**
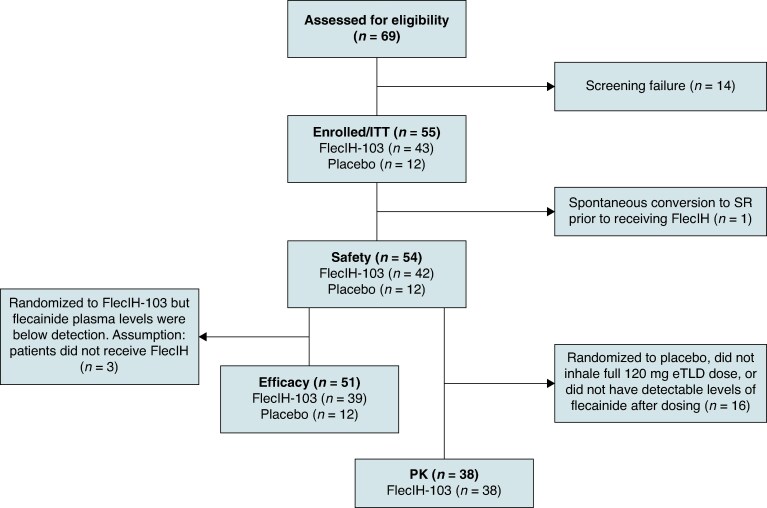
Patient disposition. Flowchart of patients assessed for trial participation, population disposition, and criteria for exclusion from study populations.

*Figure 1* provides definitions for the populations used in this study and summarizes the disposition of study subjects. From July 2022 to May 2023, a total of 69 patients consented to participate in the trial until the study was terminated and 55 patients were randomized (ITT population; FlecIH-103: *n* = 43, placebo: *n* = 12). One patient randomized to FlecIH-103 had spontaneous conversion of AF to SR prior to receiving study drug; the remaining 54 patients constituted the safety population (FlecIH-103: *n* = 42, placebo: *n* = 12).

Three patients randomized to FlecIH-103 had no detectable plasma levels of flecainide (i.e. <0.5 ng/mL) and hence were not included in the efficacy analyses. The reason for the absence of plasma levels of flecainide in these three patients was investigated, and no clear evidence was found to explain the finding. To have a more precise estimate of the efficacy of FlecIH-103, the 51 patients with plasma levels of flecainide consistent with their assigned treatment group were defined post hoc as the efficacy population (FlecIH-103: *n* = 39, placebo: *n* = 12). One patient in the FlecIH-103 group (*n* = 39) had missing blood samples and was excluded from the PK population (FlecIH-103: *n* = 38, placebo: *n* = 0).

Following the first blinded review of the data by the Data Monitoring Committee, a futility analysis was performed to estimate the probability of success (PoS) for the primary endpoint based on the data from the first 45 patients enrolled. After review of the PoS modelling, the estimated likelihood of success was too low for the study to continue and a formal decision to stop the study was made; 55 patients had been enrolled in the trial at the time it was terminated.

### Baseline characteristics

In the safety population (*n* = 54), the mean overall age was 59.6 years (SD: 13.5), 31.5% of patients were female, and 94.4% were White. At randomization, 83.3% of patients had AF symptoms for less than 24 h; 59.2% of patients were presenting with a first AF episode, 35.2% with a recurrent episode, and 5.6% with a post-ablation episode. The mean number of previous AF episodes was 1.73 (SD: 1.4). The mean CHA2DS2-VASc score was 1.4 (SD: 1.5). No statistical differences in baseline characteristics, including previous history of AF, and comorbidities were observed between treatment groups and/or conversion status. Baseline characteristics by treatment group, and conversion status are summarized in *Table 1*.

**Table 1:** Baseline characteristics and demographics

Parameter	Placebo (*n* = 12)	FlecIH-103 (safety) (*n* = 42)	FlecIH-103 (efficacy)	
			Conversion-yes (*n* = 12)	Conversion-no (*n* = 27)
Age (y), mean ± SD	64.2 ± 12.5	58.3 ± 13.6	57.4 ± 16.7	59.5 ± 13.3
Male sex, *n* (%)	7 (58.3%)	30 (71.4%)	10 (83.3%)	18 (66.7%)
White, *n* (%)	12 (100.0%)	39 (92.9%)	12 (100.0%)	24 (88.9%)
Weight (kg), mean ± SD	98.1 ± 44.7	92.6 ± 29.5	92.5 ± 37.1	94.5 ± 27.0
Height (kg), mean ± SD	159.1 ± 42.2	173.9 ± 25.9	174.2 ± 34.3	174.4 ± 23.4
Body mass index (kg/m^2^), mean ± SD	27.7 ± 2.8	26.5 ± 4.0	24.9 ± 3.5	27.2 ± 4.0
Systolic blood pressure (mmHg), mean ± SD	121.1 ± 12.9	123.7 ± 13.8	129.7 ± 17.3	120.6 ± 10.8
Diastolic blood pressure (mmHg), mean ± SD	77.5 ± 9.3	83.3 ± 8.8	84.8 ± 9.5	82.4 ± 9.0
**Medical comorbidities**				
Hypertension, *n* (%)	5 (41.7%)	9 (21.4%)	1 (8.3%)	7 (25.9%)
Hyperlipidaemia, *n* (%)	4 (33.3%)	9 (21.4%)	1 (8.3%)	8 (29.6%)
Diabetes, *n* (%)	0 (0.0%)	4 (9.5%)	1 (8.3%)	3 (11.1%)
Coronary artery disease, *n* (%)	2 (16.7%)	3 (7.1%)	1 (8.3%)	2 (7.4%)
NYHA HF Class I, *n* (%)	11 (91.7%)	41 (97.6%)	12 (100.0%)	26 (96.3%)
NYHA HF Class II, *n* (%)	1 (8.3%)	1 (2.4%)	0 (0.0%)	1 (3.7%)
CHA_2_DS_2_-VASc score, mean ± SD	2.2 ± 1.7	1.2 ± 1.3	1.1 ± 1.2	1.3 ± 1.4
**AF at presentation and previous history**				
AF duration ≤ 24 h, *n* (%)	10 (83.3%)	35 (83.3%)	11 (91.7%)	21 (77.8%)
AF symptoms duration (h), mean ± SD	15.0 ± 8.7	16.3 ± 10.3	14.5 ± 7.4	16.5 ± 11.9
Ventricular rate (bpm), mean ± SD	105.0 ± 12.0	114.7 ± 21.9	115.3 ± 22.3	115.6 ± 22.6
First AF episode, *n* (%)	5 (41.7%)	27 (64.3%)	8 (66.7%)	17 (63.0%)
Recurrent AF, *n* (%)	5 (41.7%)	14 (33.3%)	4 (33.3%)	9 (33.3%)
Post-ablation AF, *n* (%)	2 (16.7%)	1 (2.4%)	0 (0.0%)	1 (3.7%)
Previous AF episode, mean ± SD	1.4 ± 0.7	1.9 ± 1.6	2.5 ± 3.0	1.7 ± 0.9

There were no statistically significant differences (*P* > 0.05) in demographics, comorbidities, or AF history between treatment groups or when patients were compared by conversion status.

### Conversion of atrial fibrillation to sinus rhythm

**Figure 2:**
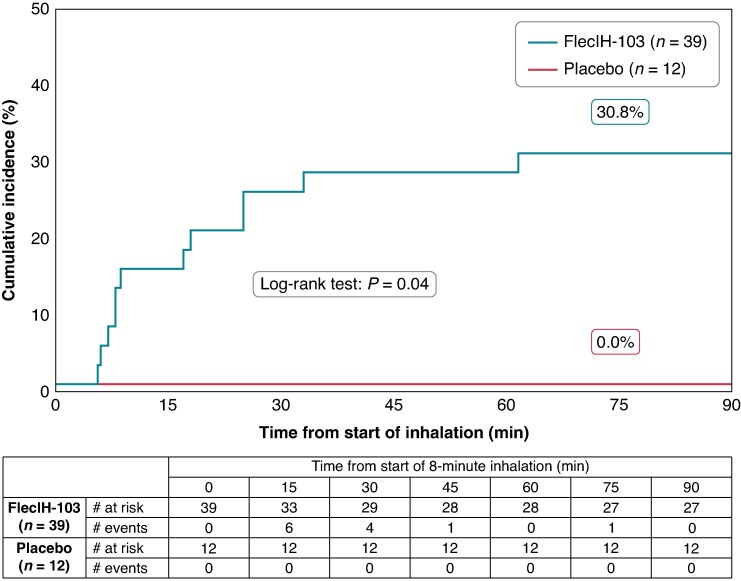
Time to conversion of AF to SR. Cumulative percentage of patients by treatment group whose AF converted to SR within the observation period. The period of inhalation comprises the first 8 min in the plot.

In the efficacy population, a total of 12 patients had their AF converted to SR within the 90 min observation period. A time-to-event curve (Kaplan–Meier curve) displays the time to conversion in the two treatment groups (*Figure 2*). Based on the KM estimator, the proportions of patients whose AF converted to SR ≤ 90 min after initiation of dosing by treatment group were 30.8% (95% CI: 14.7, 43.8) for the active group (*n* = 39) and 0.0% (95% CI: 0.0, 0.0) for the placebo group (*n* = 12; *P* = 0.04). The median time to conversion from the start of inhalation in the active group was 12.8 min (IQR: 17.2, range: 5.6, 61.6).

### Atrial fibrillation symptoms at baseline and at the end of the observation period

**Figure 3:**
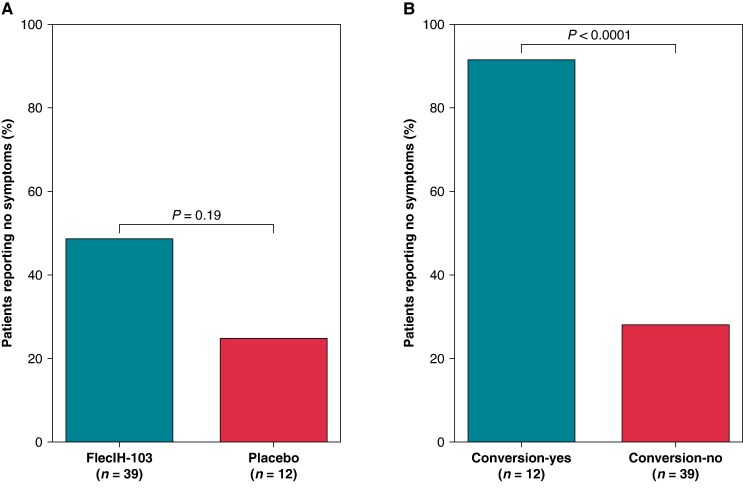
AF symptoms at 90 min. Percentage of patients who reported no symptoms at the end of the observation period based on treatment group (*A*) and conversion status (*B*).

At baseline, 98% of all patients of the efficacy population had at least one AF-related symptom (FlecIH-103: 97.4%, placebo: 100.0%). The following symptoms were reported: heart palpitations (FlecIH-103: 84.6%, placebo: 75.0%), chest pain/discomfort (FlecIH-103: 23.1%, placebo: 33.3%), dizziness/lightheadedness (FlecIH-103: 23.1%, placebo: 41.7%), and shortness of breath/dyspnoea (FlecIH-103: 20.5%, placebo: 33.3%). At the end of the 90 min observation period, 51.3% of patients in the FlecIH-103 group and 75.0% of patients in the placebo group still had at least one AF-related symptom (*P* = 0.19; *Figure 3A*). Among the 12 patients whose AF had converted to SR, only 1 (8.3%) still had an AF-related symptom compared with 71.8% of patients whose AF did not convert to SR (*P* < 0.0001; *Figure 3B*), corresponding to an 83.4% and 28.2% reduction in symptoms in the conversion-yes and conversion-no groups, respectively.

### Atrial fibrillation–related interventions

In the efficacy population (*n* = 51), a total of 30 interventions were performed prior to discharge (29 ECV, 1 PCV). In the FlecIH-103 group (*n* = 39), 20 patients (51.3%) underwent ECV or PCV, whereas in the placebo group (*n* = 12), 10 patients (83.3%) underwent ECV. Among patients whose AF did not convert to SR following study drug administration (*n* = 39), 30 patients (76.9%) required an AF-related intervention.

### Time to eligibility for discharge, hospitalizations, and follow-up

**Figure 4:**
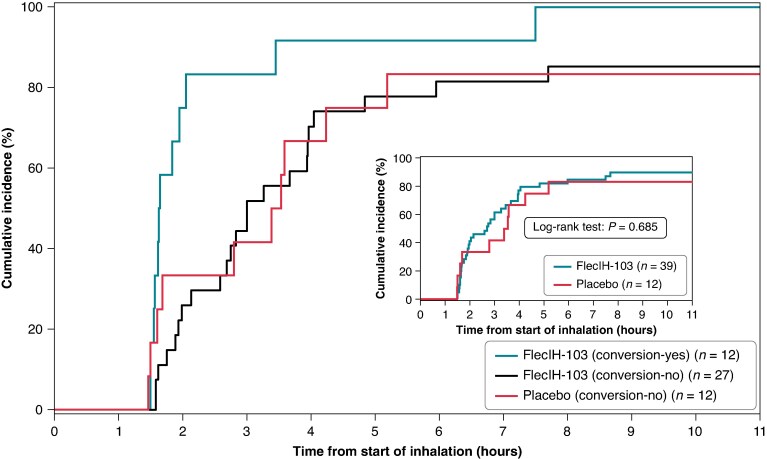
Time to discharge-eligible status by treatment group and conversion status. Cumulative percentage of patients who became eligible for discharge following drug administration for the following three groups: (1) patients who received FlecIH-103 and whose AF converted (light blue), (2) patients who received FlecIH-103 and whose AF did not convert (black), and (3) patients who received the placebo solution (red). The inset shows the time to discharge-eligible status based only on treatment group (FlecIH-103 vs. placebo).

In the efficacy population (*n* = 51), the median time to discharge-eligible status was 2.7 h for the FlecIH-103 group (*n* = 39) and 3.5 h for the placebo group (*n* = 12) (inset in *Figure 4*). When evaluated by conversion status, the median time to discharge eligibility was 1.6 h for patients whose AF converted to SR following administration of study drug (*n* = 12) and 3.3 h for those who did not convert following administration of study drug (*n* = 39) (*Figure 4*). When comparing patients in the FlecIH-103 group whose AF did not convert with those patients in the placebo group, the median time to discharge eligibility was similar (3.0 and 3.5 h, respectively), suggesting that failure to convert with FlecIH-103 does not extend the time required to be in the Emergency Department (ED) (*Figure 4*).

After the observation period, one patient in the placebo group was hospitalized for an AE of palpitations.

The presence of at least one AF-related symptom during follow-up was observed in four patients (10.5%) in the FlecIH-103 group and one patient (8.3%) in the placebo group at 24 h, while six patients (15.4%) in the FlecIH-103 and two patients (16.7%) in the placebo group reported at least one AF-related symptom at 96 h. There was no statistical significance between treatment groups for AF-related symptoms at 24 or 96 h post-dose. Only one patient, who belonged to the FlecIH-103 group, had an AF recurrence 3 days after dosing. This patient did not undergo another attempt at cardioversion and was started on metoprolol.

### Pharmacokinetics and pharmacodynamics of FlecIH-103

The mean maximum plasma concentration (*C*_max_) of flecainide in the PK population (*n* = 38) was 198 ng/mL (SD: 156), which was 1.85-fold lower than that observed for the same target dose (eTLD of 120 mg) in the INSTANT-1 study (367 ng/mL, SD: 217).^14^

Among patients in the PK population (*n* = 38), AF was converted to SR in 11 patients following FlecIH-103 administration (1 of the 12 patients in the FlecIH-103 group whose AF converted to SR had missing plasma samples and therefore was excluded from the PK population); these 11 patients had a mean *C*_max_ of 260 ng/mL (SD: 152), which was approximately 50% higher than the mean *C*_max_ of patients whose AF did not convert to SR (172 ng/mL; SD: 153).

The mean change from baseline in QRS interval duration (5 min post-dosing compared to baseline) was 3.8 ms (SD: 5.4) in the FlecIH-103 group and −3.2 ms (SD 5.2) in the placebo group. No patient had a QRS interval duration ≥ 130 ms. Vital signs other than ventricular rate did not change significantly individually over time or differ between treatment groups.

### Adverse events

In the safety population, a total of 18 (43%) patients in the FlecIH-103 group (*n* = 42) presented with a TEAE compared to 3 (25%) patients in the placebo group. There were no serious AEs or CV-AESIs reported. The most frequently reported TEAEs (≥10% of patients in either treatment group) in the FlecIH-103 and placebo groups, respectively, were as follows: dysgeusia (14.3% vs. 0.0%), dyspnoea (14.3% vs. 0.0%), cough (11.9% vs. 0.0%), and dizziness (9.5% vs. 16.7%). All treatment-emergent AEs were of mild or moderate intensity, short-lived (median: 2 h, Q1: 0.57 h, Q3: 8.3 h), and 91% resolved within 24 h of drug inhalation. Respiratory-related AEs, e.g. dyspnoea, did not affect the tolerability of FlecIH-103, and all patients were able to complete the inhalation.

## Discussion

The Phase 3, multicentre, randomized, double-blind, placebo-controlled RESTORE-1 trial sought to evaluate the efficacy and safety of FlecIH-103 compared to a placebo inhalation solution. The study was stopped early with 55 of the planned 400 patients enrolled due to the lower-than-expected conversion rate and flecainide plasma levels noted upon review of the blinded data. Despite the small sample size of 55 patients, the results show that orally inhaled flecainide at the lower-than-targeted dose (i.e. approximately 60 mg eTLD, instead of 120 mg eTLD) resulted in conversion of AF to SR in 30.8% of FlecIH-103-treated patients. While this proportion is lower than that observed in the 120 mg eTLD group in the INSTANT-1 trial [42.6% (95% CI: 33.0%, 52.6%)],^14^ it was significantly higher (*P* = 0.04) than in the placebo group (0.0%).

**Figure 5:**
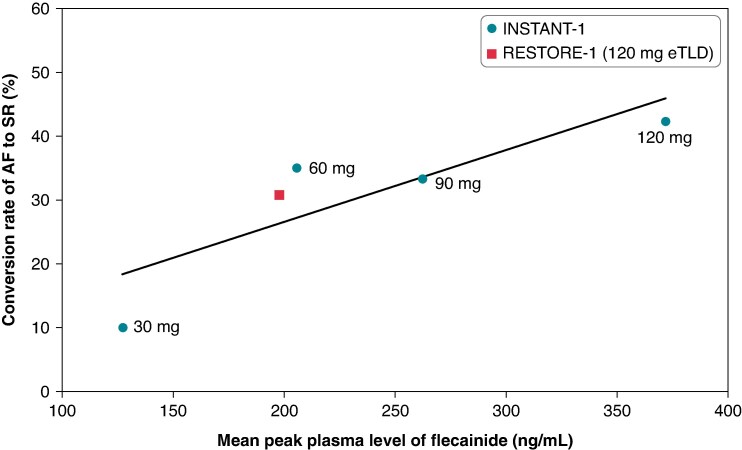
Comparison between plasma levels of flecainide and conversion rates. Plot of peak plasma levels and conversion rates from both the INSTANT-1 and RESTORE-1 trials. Each INSTANT-1 data point represents a different dose of FlecIH. The 120 mg eTLD INSTANT-1 data point is composed of patients who received two FlecIH solutions (FlecIH-102 and FlecIH-103).^13^ The data point for RESTORE-1 shows that the plasma levels achieved were similar to those of the 60 mg eTLD of INSTANT-1, which is consistent with the conversion rate in the range of 30–35%. The Pearson’s correlation coefficient was 0.85 (*P* = 0.07).

The lower-than-expected conversion rate observed in the RESTORE-1 study is consistent with the lower plasma levels achieved following administration of the 120 mg FlecIH-103 solution compared to those achieved with the same target dose in the INSTANT-1 trial (*Figure 5*). Our data suggest that the delivery rate of flecainide by the drug–device combination product used in RESTORE-1 was about half of that observed in the INSTANT-1 trial. We hypothesize that these findings were due to batch-to-batch variability in the performance of the drug–device combination product with respect to the rate of nebulization of the inhalation solution. Consequently, the total delivered flecainide dose over the 8 min inhalation period in RESTORE-1 would be about 50% of that observed in INSTANT-1 (i.e. 60 mg instead of 120 mg eTLD). Therefore, as described in the Results section, the peak plasma levels of flecainide in RESTORE-1 were similar to those observed for the 60 mg eTLD dose in the INSTANT-1 trial (*Figure 5*).^13^

Current standard treatment for recent-onset paroxysmal AF consists of either rhythm or rate control. Rhythm control can be achieved through PCV or ECV.^15^ Use of flecainide for conversion of AF to SR can be achieved with either oral administration of flecainide^16^ or via intravenous infusion. Oral flecainide is the AAD used most commonly for the ‘pill-in-the-pocket’ approach.^17^ It has been reported that a 300 mg dose of oral flecainide is associated with a conversion rate of 33–68% at 2–4 h, respectively, after dosing.^5–9^ Administration of intravenous flecainide (10 min infusion of 2 mg/kg) has been reported to have a conversion rate of 69% at 2 h after dosing^10^; however, it is associated with a higher incidence of serious AEs than both oral flecainide^9^ and inhaled flecainide.^13,14^ Among the advantages of delivering flecainide via oral inhalation, it is that it could potentially be used both under medical supervision (e.g. in-hospital setting) and via self-administration outside the hospital.

The most common symptoms reported by patients experiencing a new-onset episode of AF are palpitations, dyspnoea, chest pain, and fatigue,^18^ and these symptoms and their prevalence were similar in the current study. The presence of AF-related symptoms is considered by patients as negatively affecting their quality of life,^19^ highlighting the importance of restoring SR (rhythm control therapy) or slowing ventricular rate (rate control therapy). In both the RESTORE-1 trial and the INSTANT-1 trial,^14^ the restoration of SR after administration of FlecIH-103 was associated with significant reduction or resolution of symptoms. In fact, among the patients in RESTORE-1 whose AF was converted to SR, the incidence of symptoms was reduced by 83.4%.

Patients whose AF was successfully converted to SR with FlecIH-103 were eligible for discharge sooner than those whose AF did not convert (1.6 h vs. 3.3 h; *P* = 0.002) (*Figure 4*). When comparing patients who received FlecIH-103 and whose AF did not convert with those in the placebo group, the time to discharge eligibility was not significantly different (3.0 h vs. 3.3 h; *P* = 0.280) (*Figure 4*). This suggests that having an unsuccessful attempt at conversion with FlecIH-103 did not result in further delay in patient management (e.g. ECV) or prolong the time the patient spent in the ED, although this hypothesis should be prospectively evaluated. In summary, these data demonstrate that when AF is successfully converted to SR, symptoms are reduced or abolished, and vital signs normalize and become stable, resulting in early discharge, whereas failure to cardiovert with FlecIH-103 did not delay discharge from the ED further than for those who received placebo.

Plasma levels of flecainide have a wide therapeutic range (200–1000 ng/mL),^20^ yet the relationship between plasma levels and conversion of recent-onset AF to SR has not been well-established. In a study by Suttorp *et al*. and in the previous INSTANT-1 study,^14,21^ the comparison between patients whose AF converted to SR vs. those whose AF did not convert did not show significant differences in flecainide plasma levels between the two groups. Yet, when analysing the correlation between plasma levels and conversion rates based on the dose-escalation INSTANT-1 study (Part A)^13^ and now in the RESTORE-1 trial, a correlation between plasma levels and conversion rates was observed (*Figure 5*).

The use of oral inhalation as the route to deliver flecainide is most commonly associated with respiratory symptoms, such as cough, oropharyngeal discomfort, and hoarseness.^13,14^ In the current study, AEs were all mild or moderate in intensity and of short duration. Cardiac events of special interest that were associated with flecainide administration, such as hypotension and AFL, did not occur in this study, possibly because of lower plasma levels achieved. Consistent with the safety profile observed in the INSTANT-1 study, coughing during inhalation was not significantly correlated with either flecainide plasma levels or conversion rates.

## Limitations

The main limitation of the current study was the early termination of the trial due to the lower-than-expected conversion rates and peak plasma levels noted during blinded data review. Despite the loss of statistical power due to the early termination, the log-rank test showed a significant difference between the active and placebo treatment groups (*P* = 0.04). However, the small sample size (*n* = 55) and the fact that there were zero conversions in the placebo group, which is lower than reported in the literature (∼5%),^22,23^ limit the interpretation of the results of the study.

The conversion rate for the same target dose of FlecIH-103 (i.e. an eTLD of 120 mg) in the Phase 2 INSTANT-1 study^14^ was substantially higher (42.6%) than that observed in the current study (30.8%). This difference in conversion rate could be explained by the data shown in *Figure 5*, that is, due to the lower flecainide plasma levels achieved in RESTORE-1 (198 ± 156 ng/mL) vs. INSTANT (367 ± 217 ng/mL). As described above, we hypothesize that these findings were due to batch-to-batch variability in the performance of the drug–device combination product with respect to the rate of nebulization of the inhalation solution.

## Conclusions

Despite early termination of this study, the results of the RESTORE-1 study are consistent with the efficacy and safety of inhaled flecainide observed in the Phase 2 trial and warrant further clinical development of FlecIH-103 for the conversion of recent-onset AF to SR. The PK data suggest that drug delivery was approximately half of the target level, providing an explanation for the lower-than-expected conversion rate observed in the active treatment arm.

Future studies should focus on improving the consistency and reliability of FlecIH-103 delivery while maintaining safety. Towards this goal, in an ongoing Phase 1 study, we are testing delivery of our inhaled flecainide formulation using a novel investigational vibrating mesh nebulizer. This drug–device combination product may deliver flecainide more efficiently and consistently to the lung. In addition, a dosing regimen that includes the administration of a booster dose is being investigated to further increase efficacy.

## Data Availability

All data requests will be made through the corresponding author. Data requests will require details of the analysis and usage to be performed and will need to be approved by the study steering committee. If approved and within the confines of the Human Research Ethics approval, data will be made available.
